# Endobronchial Ultrasound-Guided Transbronchial Needle Aspiration (EBUS-TBNA): Technical Updates and Pathological Yield

**DOI:** 10.3390/diagnostics11122331

**Published:** 2021-12-10

**Authors:** Huzaifa A. Jaliawala, Samid M. Farooqui, Kassem Harris, Tony Abdo, Jean I. Keddissi, Houssein A. Youness

**Affiliations:** 1 Interventional Pulmonary Program, Section of Pulmonary, Critical Care and Sleep Medicine, The Oklahoma City VA Health Care System, The University of Oklahoma Health Sciences Center, Oklahoma City, OK 73104, USA; Huzaifa-Jaliawala@ouhsc.edu (H.A.J.); Samid-Farooqui@ouhsc.edu (S.M.F.); Tony-Abdo@ouhsc.edu (T.A.); jean-keddissi@ouhsc.edu (J.I.K.); 2 Division of Pulmonary and Critical Care Medicine, Section of Interventional Pulmonology, Westchester Medical Center, Valhalla, NY 10595, USA; Kassem.Harris@wmchealth.org

**Keywords:** endobronchial ultrasound, transbronchial needle aspiration, diagnostic yield

## Abstract

Since the endobronchial ultrasound bronchoscope was introduced to clinical practice, endobronchial ultrasound-guided transbronchial needle aspiration (EBUS-TBNA) has become the procedure of choice to sample hilar and mediastinal adenopathy. Multiple studies have been conducted in the last two decades to look at the different technical aspects of the procedure and their effects on the final cytopathological yield. In addition, newer modes of ultrasound scanning and newer tools with the potential to optimize the selection and sampling of the target lymph node have been introduced. These have the potential to reduce the number of passes, reduce the procedure time, and increase the diagnostic yield, especially in rare tumors and benign diseases. Herein, we review the latest updates related to the technical aspects of EBUS-TBNA and their effects on the final cytopathological yield in malignant and benign diseases.

## 1. Introduction

Over the last two decades, there have been major developments in our ability to sample mediastinal and peribronchial structures. We have come a long way from the initial use of transbronchial needle aspiration (TBNA) for sampling through a rigid bronchoscope in 1949, to Wang et al. introducing TBNA through flexible bronchoscopy in 1983. Endobronchial ultrasound (EBUS) with real-time visualization of mediastinal structures was first employed in 1992 [1]. As pulmonologists gain more experience in performing EBUS-TBNA, the ability to diagnose pathological involvement of mediastinal structures has significantly improved. The American College of Chest Physicians (ACCP) guidelines for non-small cell lung cancer (NSCLC) report that EBUS-TBNA has a 91% sensitivity in establishing a diagnosis as compared with 81% for cervical mediastinoscopy [2]. Given EBUS-TBNA’s minimally invasive nature and high sensitivity, it has become the procedure of choice to stage the mediastinum [3].

Multiple studies have evaluated different components of the EBUS-TBNA procedure to optimize the pathological yield and eliminate unnecessary steps while reducing the time and complications. Newer ultrasound modes and many bronchoscopic tools have been introduced to optimize the selection of the lymph node and the collection of cytological and histological material.

In this article, we aim to review the latest updates related to the technical performance of EBUS-TBNA and describe how emergent data can affect its diagnostic yield in lung cancer. In addition, we discuss the utility of EBUS-TBNA in the diagnosis of lymphoma and sarcoidosis.

## 2. Procedure-Related Technical Factors and Their Effects on Diagnostic Yield

### 2.1. Choice of Sedation

EBUS-TBNA is usually performed in the endoscopy suite under moderate sedation (MS), deep sedation, or general anesthesia (GA) [4]. The choice of anesthesia strategy is largely driven by institutional policy and operator preference. In a prospective randomized study, Casal et al. compared the diagnostic yield of EBUS-TBNA performed under MS with GA. There was no difference in the diagnostic yield, the number of lymph nodes (LN) sampled, the number of passes per LN, or the rate of major complications. It should be noted that 6% of patients assigned to the MS group did not tolerate sedation, and the EBUS-TBNA had to be done under GA [5].

### 2.2. Needle Size

The optimal needle size has been a subject of interest for interventional pulmonologists since the introduction of EBUS-TBNA. The factors that need to be considered are the ability to acquire satisfactory specimens to establish the diagnosis without increasing the side effects. The current literature supports the use of 21 G or 22 G needles, as no difference in specimen adequacy or diagnostic yield was found between the two sizes [6].

When compared with the 22 G needle in sampling the same LN, the 25 G needle had a similar diagnostic yield for malignancy. The histology specimens containing malignant cells and the number of malignant cells were significantly higher in the 22 G compared with the 25 G needle, and no difference in complications was seen [7,8]. Other studies comparing aspirate done with 25 G and 22 G needles showed comparable specimen adequacy and diagnostic accuracy [9]. Similarly, there was no difference in specimen adequacy and diagnostic yield when the 25 G needle was compared with the 21 G needle [10].

Moreover, when the 22 G was compared with the larger 19 G needle in sampling the same LN in an alternating manner, there was no improvement in the overall diagnostic yield. However, more bloody passes and lower sample adequacy were observed with the 19 G needle aspirates [11]. Similarly, in a randomized controlled trial of 78 patients, Dooms et al. showed that despite having a larger tissue aspirate, the specimen was bloodier with the 19 G needle, with an overall similar diagnostic yield and specimen quality compared with the 22 G needle [12].

In a prospective analysis of 83 EBUS-TBNA samples obtained from 47 patients, sampling of the same LN with 19 G and 21 G needles showed more cellular material based on the cell area in the cell block obtained with the 19 G compared with the 21 G (7.34 vs. 5.23 mm^2^, *p* = 0.02) [13]. In a prospective randomized controlled trial that included 107 patients, Wolters et al. showed that aspirates using the 19 G needle contained significantly more tissue and tumor cells compared with the 22 G needle [14]. However, it remains unclear whether this difference affects the molecular analyses and PD-L1 staining of these specimens.

Conversely, in a retrospective single-center study, Jones et al. found a higher proportion of lymphoma (9%, 5%, and 0%) and benign disease (89%, 70%, and 38%) in LN sampled with the 19 G, 21 G, and 22 G, respectively. The 19 G needle was observed to be superior to both 21 G and 22 G in subclassifying malignant diseases, with lower rates of NSCLC-NOS (non-small cell lung cancer—not otherwise specified), and it reduced the need for invasive mediastinoscopy [15].

Although studies comparing different needle sizes did not show any statistically significant differences in diagnostic yield, we cannot exclude that a small difference may exist, especially as some of these studies were underpowered to detect a difference, had a retrospective design, and may not have tested different needles on the same lymph nodes (Table 1).

### 2.3. Use of Suction and Stylet

EBUS-TBNA has traditionally been performed with the needle advanced and with a stylet occluding the needle until the lymph node is accessed under EBUS guidance. A 20 cm suction is then applied, and aspiration is done by moving the needle in the lymph node 10 to 20 times. The addition of suction has not been shown to improve the diagnostic yield or sample adequacy when compared with lower suction of 10 cm or no suction at all [16,17,18].

In a randomized controlled trial, Lin et al. evaluated the diagnostic yield of malignancy and the specimen adequacy of using suction and a stylet, suction with no stylet, and stylet with no suction. Each LN was sampled with the three methods using a 22 G needle. There were no significant differences among the groups in specimen adequacy rate or diagnostic yield of malignancy, although using suction increased the tissue-core acquisition rate compared with the no suction group [19].

### 2.4. Fanning

Fanning is a technique employed by endoscopists to influence the diagnostic yield of a procedure. It consists of sampling multiple areas within a lymph node in each pass by altering the angle of the needle with each subsequent agitation during a pass. This was shown to be superior in EUS-FNA of pancreatic lesions [20], but no such data are present for EBUS-TBNA. However, preliminary data involving lymph nodes in ex vivo calf lungs have shown that fanning methods collected larger samples as compared with no fanning [21].

### 2.5. Core Needle

**Figure 1:**
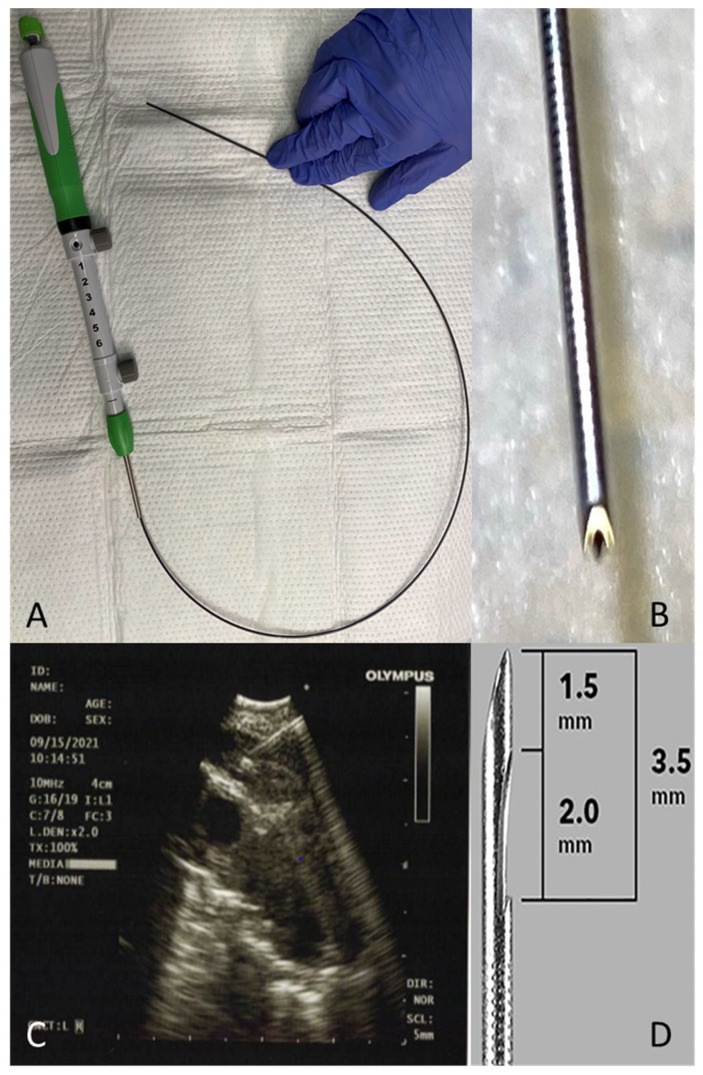
Core biopsy needle. (**A**) Acquire^®^ 22 G FNB needle. (**B**) Acquire Franseen needle tip. (**C**) EBUS-FNB of a hilar lymph node. (**D**) ProCore^®^ needle with reverse bevel (image obtained with permission from John Wiley & Sons, Inc., Hoboken, NJ, USA) [24].

Core biopsy specimens can also be obtained via EBUS. These samples involve the use of a Franseen tip 22 G fine needle biopsy (FNB) device equipped with three cutting edges (Acquire^®^ 22 G FNB needle, Boston Scientific Co., Natick, MA, USA) (Figure 1A–C). In a study evaluating the diagnostic yield of FNB in EUS compared with a historical control using the Expect^®^ 22 G FNA needle (Boston Scientific Co., Natick, MA, USA), FNB had better histological samples in fewer attempts [22]. In a retrospective analysis of the first 100 patients undergoing EBUS with FNB, Balwan et al. showed that core biopsy was seen in 87% of patients, the pathological diagnosis was established in 97%, and the diagnostic yield for granulomatous lymphadenopathy was obtained in 95.6%. No patient-related adverse events were noted [23].

The ProCore^®^ needle from Cook Medical (Bloomington, IN, USA) is designed to provide a core of histological tissue in contrast to the cytological specimens from standard fine needle aspirations. It comes in two sizes, 22 G and 25 G. It has a reverse bevel that aims to collect a core histological sample by shearing material from the lesion during retrograde motion (Figure 1D) [24].

In a retrospective study comparing 110 patients who had an EBUS using a 22 G needle with 125 patients who had an EBUS using the ProCore^®^ needle, the EBUS core biopsy had a higher sensitivity than standard EBUS-TBNA (92% vs. 77%, *p* = 0.001). Additional sampling methods such as mediastinoscopy and CT-guided FNA were obtained in 30% of patients who underwent standard EBUS-TBNA versus 15% of those who had EBUS core biopsy (*p* = 0.006) [25]. However, in a prospective trial, Dhooria et al. found no difference in the diagnostic yield of patients with intrathoracic lymphadenopathy with suspected sarcoidosis when these patients were randomized to EBUS-TBNA with the ProCore needle versus the standard 22 G TBNA needle [26].

### 2.6. Mini-Forceps Biopsy

**Figure 2:**
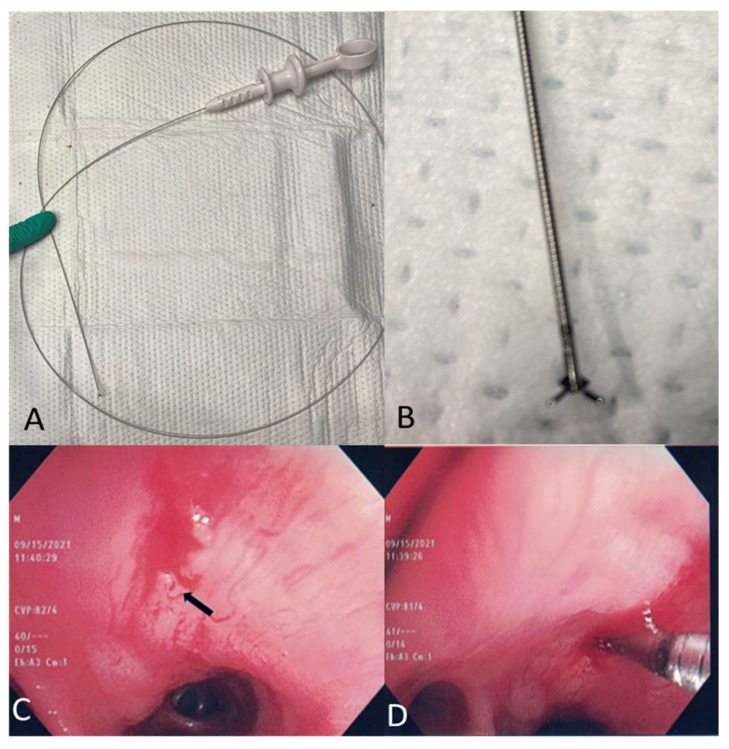
Mini-biopsy forceps. (**A**) Boston Scientific CoreDxTM Pulmonary Mini-Forceps. (**B**) Tip of the CoreDxTM Pulmonary Mini-Forceps. (**C**) Hole (arrow) created in the mucosa by TBNA needle. (**D**) Mini-forceps passed through the hole for biopsy of mediastinal structures.

A histological sampling of the lymph node can be done via mini-biopsy forceps, which is introduced through the initial hole made by the TBNA needle (Figure 2A–D).

Herth et al. evaluated the role of transbronchial forceps biopsy (TBFB) in 75 patients without known or suspected NSCLC. Specimens were acquired from subcarinal lymph nodes larger than 2.5 cm. Sampling was done with a 22 G needle, 19 G needle, and a 1.15 mm (FB-56D-I; Olympus Ltd., Japan) mini-forceps with a cup opening of 7.3 mm. A diagnosis was obtained in 36%, 49%, and 88% of the cases while using the 22 G needle, 19 G needle, and the mini-forceps, respectively. The mini-forceps diagnostic yield compared with the needle was the highest in patients with sarcoidosis (88% vs. 36%, *p* = 0.001) and lymphoma (81% vs. 35%, *p* = 0.038). No complications occurred [27].

Similarly, Chrissian et al. showed that combining EBUS-TBNA with EBUS-TBFB in a population of 50 patients with a low likelihood of NSCLC resulted in a higher diagnostic yield of 97% compared with either modality alone (81% for EBUS-TBNA and 91% for EBUS-TBFB), with no additional complications [28].

In a retrospective study of 91 patients who had a forceps biopsy with EBUS-TBFB after a non-diagnostic rapid on-site evaluation (ROSE), no difference was seen in the overall diagnostic yield of TBNA versus TBFB. Out of the non-diagnostic TBNA samples on rapid on side evaluation (ROSE) and cell block, subsequent TBFB sampling resulted in additional pathological diagnosis in 16% of the cases; 67% of these were non-caseating granulomas. No complications were reported [29].

A meta-analysis of six observational studies included 443 patients in whom TBFB was performed after the initial EBUS-TBNA. Comparing EBUS-TBNA + EBUS-TBFB vs. EBUS-TBNA alone, the pooled overall diagnostic yield was 92% vs. 67% (*p* < 0.00001), the diagnostic yield for sarcoidosis was 93% vs. 58% (*p* < 0.00001), and the diagnostic yield for lymphoma was 86% vs. 30% (*p* = 0.03). Pneumomediastinum occurred in 1%, pneumothorax in 1%, and bleeding in 0.8% of the patients [30].

Currently available mini-forceps include the Olympus mini-forceps (FB-56D-I; Olympus Ltd., Tokyo, Japan) with an outer diameter of 1.15 mm and a cup opening of 7.3 mm [27], the Boston Scientific “SpyBite biopsy” Forceps (model: M00546270, Natick, MA, USA) with an outer diameter of 1 mm [29], and the CoreDx™ Pulmonary Mini-Forceps by Boston Scientific with an outer diameter of 0.96 mm and a 4.3 mm jaw opening (Figure 2). The specimen obtained by EBUS-TBFB should be handled as the histology specimen and placed in formalin for fixation or in saline if culture is required [31].

In summary, EBUS-TBFB appears to be complimentary to EBUS-TBNA and can be used when additional tissue is needed for molecular marker studies and in the diagnosis of lesions when the initial sampling with EBUS-TBNA is inadequate or non-diagnostic.

### 2.7. Lymph Node Cryobiopsy under EBUS Guidance

A histological sampling of the lymph node has been reported with the use of a 1.1 mm cryobiopsy probe placed in the same hole created by the TBNA with a 3 sec freeze time before pulling the probe out. The specimen is thawed in saline and fixed in formalin [32]. In a prospective study of 197 patients undergoing EBUS-TBNA and EBUS cryobiopsy for mediastinal lesions of at least 1 cm, cryobiopsy had higher sensitivity than TBNA in rare tumors (91% vs. 25%; *p* = 0.001) and benign disorders (81% vs. 53%; *p* = 0.04). The diagnostic yield was similar in malignant lymphadenopathy. Two cases of pneumothorax and one case of pneumomediastinum were reported [33].

### 2.8. EBUS Elastography

**Figure 3:**
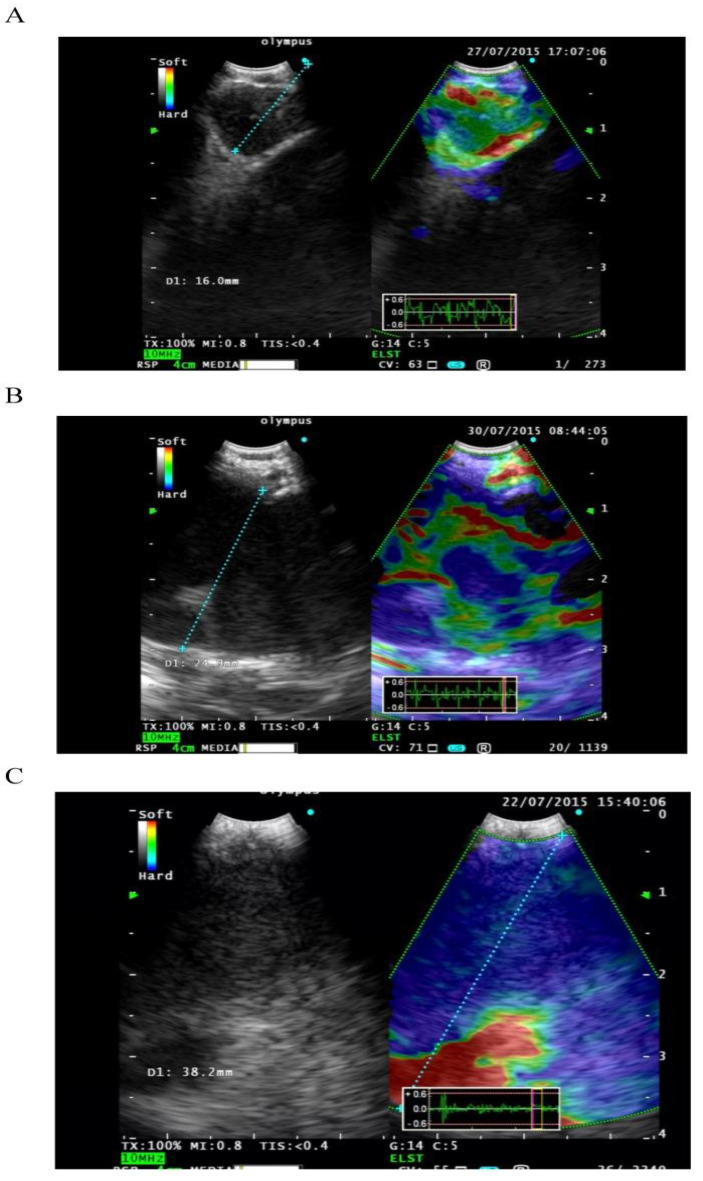
Elastography. (**A**) Type 1; predominantly non-blue (green, yellow, and red). (**B**) Type 2; part blue, part non-blue (green, yellow, and red). (**C**) Type 3 predominantly blue (reproduced with permission from Reference [37]).

Pathological processes make the tissue harder compared with the normal surrounding structure. Elastography is a recent modality that allows the calculation and visualization of tissue elasticity during EBUS. Data are converted into an RGB (red, green, and blue) color image where hard tissue is shown in blue, medium tissue in green, and soft tissue in red [34]. The images are then superimposed onto the standard grayscale B-mode ultrasound scan [35]. Lesions can be classified as type I, predominantly non-blue; type II, partly blue; and type III, predominantly blue (Figure 3). Types I and III (but not type II) were shown to be highly accurate in predicting benign or malignant disease, respectively [34,36,37].

Quantitative elastography data can also be produced by measuring the strain ratio (SR) of the lesion compared with the normal surrounding tissue. Malignant lymph nodes have a higher SR. An SR > 2.5 had a 100% sensitivity for predicting malignant lymph nodes [34].

A meta-analysis of 17 studies for differentiating benign versus malignant adenopathy found a pooled sensitivity of 0.90 (95% confidence interval (CI), 0.84–0.94) and specificity of 0.78 (95% CI, 0.74–0.81) [38], suggesting that this modality could be important in real-time differentiation of benign versus malignant lymphadenopathy.

## 3. Evaluation and Processing of Cytopathological Material Obtained by EBUS

### 3.1. Rapid On-Site Evaluation

ROSE of EBUS-TBNA samples allows for the rapid evaluation of the adequacy of the sample and a preliminary diagnosis before the specimen is completely evaluated. ROSE helps in assessing the adequacy of the sample, determining the need for additional molecular testing, and potentially decreasing the number of procedural sites. Multiple studies have been performed to compare the utility of ROSE for EBUS-TBNA samples [39,40,41,42]. Griffin et al. demonstrated, in a retrospective study, that there was no difference in diagnostic yield or number of sampled sites with the use of ROSE [39]. In a prospective small study including 81 patients who underwent EBUS with and without ROSE, Cardoso et al. found that the use of ROSE resulted in a higher rate of adequate samples and diagnostic accuracy, although the difference did not reach statistical significance [42]. In a larger randomized prospective trial of 236 patients undergoing EBUS-TBNA, the diagnostic yield in the ROSE group was significantly higher (90% vs. 81%, *p* = 0.003) and the rate of a suspicious specimen on cytology and non-diagnostic specimen in pathology was significantly lower compared with the non-ROSE group [43]. Another randomized controlled trial of 108 patients showed that ROSE use resulted in a lower puncture number with no increase in the procedure time. Even though the overall diagnostic yield was higher in the ROSE group (85% vs. 75%), it did not reach statistical significance [44].

Overall, ROSE is a helpful tool to optimize the preparation of the specimen and evaluate the adequacy of lymph node sampling. It may help in increasing the diagnostic yield and avoiding repeated procedures (for additional desired testing) without affecting the total time of the procedure [45].

### 3.2. Adequacy of Samples Obtained during EBUS

Currently, there are no defined standardized criteria to determine the adequacy of a sample obtained with EBUS. In general, a sample is considered adequate when a diagnosis is made, such as granuloma or malignancy even in the absence of lymphoid tissue, or if sufficient benign lymphoid tissue is present [45].

Nayak et al. defined an adequate sample as any smear that contains more than 5 fields with at least 100 lymphocytes per low-power field (×100) in a smear PLUS less than 2 groups of bronchial cells per low-power field (×100), or the presence of germinal center fragments, irrespective of the above-mentioned criteria [46]. In addition, any smear with positive results such as malignancy or granuloma was considered adequate. Based on the above, each site can be assigned one of the following categories: non-diagnostic, negative for the disease, granulomatous, suspicious for malignancy, or positive for malignancy [46]. In 2016, Choi et al. suggested using a core tissue length of at least 2 cm, presence of malignant cells, anthracotic pigment, or a lymphocyte density of more than 40 per 10 high-power fields (at ×40 magnification) as criteria for an adequate specimen [47].

### 3.3. Cell Block and Molecular Testing

Cell block is a technique to preserve gross pathological specimens using paraffin blocks for histopathological analysis. It allows for the evaluation of cytological architecture and immunochemical staining, thereby allowing for better characterization of the malignancy [48]. Cell block analysis can increase the yield of EBUS-TBNA by 7% and can generate data for genetic analysis in patients with adenocarcinoma [49]. In 2008, Lee et al. found that a maximum of three passes per lymph node station resulted in a maximal yield for cytopathologic diagnosis [50].

The identification of predictive malignant cell biomarkers in NSCLC has enabled targeted therapies to be utilized for better patient outcomes [51]. Testing for tumor markers such as EGFR, KRAS, and ALK has hence become the standard of care [52]. Once a diagnosis of carcinoma is obtained, extra passes for a cell block should be done for additional studies [45].

In a retrospective study from 2013 of 85 patients who underwent EBUS-TBNA, Yarmus et al. showed that a minimum of four passes per lymph node station is required to provide an adequate amount of specimen when advanced molecular marker analysis is limited to EGFR, KRAS sequencing, and *ALK* fluorescence in situ hybridization [53]. With the increased availability of additional targetable biomarkers to drive treatment decisions, it remains unclear what is the optimal number of passes that should be obtained [45].

EBUS-TBNA can provide adequate DNA sampling for next-generation sequencing (NGS). The amount of DNA needed for this modality depends on the NGS technique used. Cho et al. found the average total DNA amount from EBUS sampling to be 1971 ng with a range of 100 ng to 10,340 ng [54].

In general, the adequacy of EBUS-TBNA for molecular analysis depends on the sample size, cellularity, tumor cell fraction in the samples, the presence of contaminants such as blood or bronchial cells, and the sensitivity of the molecular testing platform [45,55]. Trisolini et al. evaluated the role of ROSE in molecular profiling in NSCLC and found that complete genotyping was achieved in 90% of the ROSE arm compared with 80% of the non-ROSE arm [56]. Although this difference did not reach statistical significance, it may be clinically relevant. A close collaboration between the molecular lab and the cytopathologist is required to determine sample adequacy for molecular testing.

Overall, the recent literature shows that lymph node sampling via EBUS-TBNA can provide enough material at least 92% of the time for a complete genomic test, which included several biomarkers by NGS and nCounter [57], and more than 94% of the time for immunohistochemical testing for PD-L1 [57,58].

## 4. Optimizing the Diagnostic Yield of EBUS in Lymphoma and Sarcoidosis

### 4.1. Lymphoma

EBUS-TBNA is a relatively safe procedure for the evaluation of mediastinal/hilar lymphadenopathy, with a reported diagnostic yield of up to 90% [59]. That yield is lower for lymphoma. Studies looking at the yield of EBUS-TBNA for lymphoma have included very low numbers of patients.

In one of the largest studies, which included 75 patients with a final diagnosis of lymphoma, EBUS-TBNA was able to establish a diagnosis in 84% of the patients and was able to subtype lymphoma in 67% of de novo cases and in 81% of the relapsed cases [60]. The lowest yield was in patients with Hodgkin’s lymphoma compared with non-Hodgkin’s lymphoma [60], and in newly diagnosed compared with recurrent lymphoma [60,61]. Moonim et al. prospectively reviewed 100 cases of suspected lymphoma sampled by EBUS-TBNA. A final diagnosis was achieved in 88% of the de novo lymphoma cases and 100% of the relapsed cases. The reported diagnostic accuracy was 91% with the lowest sensitivity (79%) reported for Hodgkin’s lymphoma [62]. Dayan et al. reported similar results with a diagnostic accuracy of 92% [63].

While EBUS-TBNA can be the first diagnostic modality, EBUS-TBFB and EBUS core biopsies might be able to provide larger histopathological tissue to help in subtyping lymphoma [27,28]. In one meta-analysis of 443 patients, adding mini-forceps biopsy to EBUS-TBNA increased the diagnostic yield significantly from 30% to 86% (*p* = 0.03) [30].

Flow cytometry is of particular importance in the immunological phenotyping of lymphomas [64]. Since diagnosis and subtyping are essential to provide treatment for patients with lymphoma, negative results should not exclude lymphoma [61]. Surgical excision remains the gold standard [65].

### 4.2. Sarcoidosis

EBUS-TBNA is the first choice for pathological confirmation of sarcoidosis [66]. It has a pooled sensitivity of more than 80% for diagnosing sarcoidosis [67], significantly higher than transbronchial lung biopsy (TBB) or endobronchial biopsy (EBB) alone. The combination of EBUS-TBNA with TBB and EBB results in a significant increase in the diagnostic yield (90%) for the diagnosis of stages I and II sarcoidosis [68,69,70]. In addition to improving the diagnostic yield, EBUS-TBNA with rapid on-site evaluation may alleviate the need to perform unnecessary TBB [71].

In a prospective study of 109 patients who underwent EBUS-TBNA for suspected stages I and II sarcoidosis, the cumulative yields for detecting non-caseating granulomas through the first, second, third, fourth, fifth, and sixth passes for the main target lesion were 63%, 75%, 82%, 85%, 86%, and 88%, respectively. The increase was statistically significant up to pass #4 [72]. A higher yield was associated with sampling nodes with a short axis of more than 1 cm, and with stage I compared with stage II sarcoidosis [73]. The number of nodes sampled appears to increase the yield in some studies [74], but not in others [73].

Procedurally, the most common endosonographic findings from sarcoidosis lymph nodes include the presence of a homogeneous texture, oval shape, a conglomeration of lymph nodes [75,76], distinct margins [75,76], and increased non-hilar perfusion [76]. The presence of the necrosis sign and absence of the clustered formation were independent factors predictive of tuberculous nodes as opposed to sarcoidosis [76].

There was no difference in the diagnostic yield in relation to needle size (22 G vs. 25 G [9], or 21 G vs. 22 G [77]) in patients suspected of having sarcoidosis. Similarly, the number of agitations of the EBUS needle (10 vs. 20) did not influence the diagnostic yield or the specimen adequacy in this population [78]. However, adding mini-forceps biopsy to EBUS-TBNA increased the diagnostic yield significantly from 58% to 93% (*p* < 0.00001) in one meta-analysis of 443 patients [30].

## 5. Conclusions

EBUS-TBNA remains the first-line minimally invasive test to evaluate mediastinal and hilar adenopathy. Since its introduction, the procedure has been refined to eliminate unnecessary steps and reduce the procedure time while optimizing the diagnostic yield. In areas where no difference between the different techniques was found, larger high-quality randomized controlled trials are recommended. In addition to providing nodal staging, EBUS-TBNA allows the acquisition of molecular markers that are essential in guiding the choice of therapy in patients with non-small cell lung cancer. It also provides an excellent diagnostic yield in stages I and II sarcoidosis. Newer tools such as core needles and mini-forceps are now available, and they appear to increase the histopathological specimen size and possibly the diagnostic yield in patients with lymphoma and benign diseases, therefore reducing the need for more invasive interventions such as mediastinoscopy.

**Table 1:** Summary of the effects of different interventions on the diagnostic yield of EBUS. LN = lymph node. * Trials comparing different techniques on the same LN.

Intervention	Type of Study and Number of Patients/LN (*n*)	Overall Findings
Needle size21 vs. 22 G	Retrospective study (*n* = 1235 patients) [6] Systematic review [2]	No statistically significant difference in diagnostic yield
Needle size22 vs. 25 G	Prospective randomized crossover study (*n* = 102 patients) [7] Retrospective propensity-matched study (*n* = 158 LN) [9]	No statistically significant difference in diagnostic yield
Needle size21 vs. 25 G	* Prospective study (*n* = 50 patients) [10]	No statistically significant difference in diagnostic yield
Needle size22 vs. 19 G	* Single-center prospective study (*n* = 27) [11] Randomized controlled trial (*n* = 78) [12] Prospective randomized trial (*n* = 107 patients) [14]	No statistically significant difference in diagnostic yield More bloody passes and lower sample adequacy were observed with the 19 G needle aspirates More cellular material in the cell block obtained with the 19 G
Needle size21 vs. 19 G	* Prospective study (*n* = 47 patients) [13]	No statistically significant difference in diagnostic yield More cellular material in the cell block obtained with the 19 G
Needle size19 vs. 21 vs. 22 G	Retrospective study (*n* = 300 patients) [15]	A higher proportion of lymphoma and benign disease found in LN sampled with the 19 G compared with 21 G and 22 G
Stylet use and suction	Prospective, randomized, non-inferiority trial comparing no suction to 10 mL and 20 mL of suction (*n* = 323 lymph node) [16] * Prospective, non-inferiority study of suction versus no suction (*n* = 26 patients) [17] Prospective randomized trial of suction versus no suction (*n* = 115 patients) [18] * Randomized controlled trial comparing suction–stylet, suction–no stylet, and stylet–no suction (*n* = 97 patients) [19]	No statistically significant difference in diagnostic yield Suction increased core tissue acquisition rate compared with no suction
Fanning vs. no fanning	Sample study on ex vivo tissue models (*n* = 18 targets) [21]	Fanning collected larger samples
Core needle versus 22 G	Retrospective study (*n* = 235 patients) [25]	Core needle biopsy had higher overall sensitivity compared with standard EBUS-TBNA. Additional biopsy tests such as mediastinoscopy and CT-guided FNA were obtained in fewer patients who underwent EBUS core biopsy.
Number of passes	Prospective study (*n* = 102 patients) [50]	A maximum of three passes per lymph node station results in a maximal yield for cytopathologic diagnosis.
ROSE vs. no ROSE	Retrospective study (*n* = 294 EBUS specimens) [39] Randomized controlled trial (*n* = 236 patients) [43]	Conflicting studies about the diagnostic yield ROSE resulted in a lower puncture number with no increase in the procedure time
Cell block	Retrospective review (*n* = 85 patients) [53]	A minimum of four passes per lymph node station is required for cell block analysis
Mini-forceps vs. TBNA	* Prospective study evaluating 22 G, 19 G, and mini-forceps biopsy (*n* = 75) [27] * Prospective study (*n* = 50 patients) [28]	Mini-forceps resulted in a higher diagnostic yield in sarcoidosis and lymphoma
Lymph node cryobiopsy vs. TBNA	* Randomized controlled trial (n = 197 patients) [33]	Cryobiopsy had a higher sensitivity in benign but not in malignant lymphadenopathy
Elastography	Prospective study (*n* = 120 LN) [34] Retrospective study (*n* = 75 LN) [36] Retrospective study (*n* = 78 LN) [37]	High correlation of non-blue and predominantly blue lesions with benign and malignant diseases, respectively
